# Risk factors and colonization status of methicillin-resistant *Staphylococcus aureus* among newly admitted newborns in neonatial intensive care unit

**DOI:** 10.1186/1753-6561-5-S6-P6

**Published:** 2011-06-29

**Authors:** JH Choi, HK Ki, JS Jeong

**Affiliations:** 1 Infection control, Konkuk University Hospital, Korea, Republic Of; 2 Department of Clinical Nursing, University of Ulsan, Seoul, Korea, Republic Of

## Introduction / objectives

To determine the prevalence of methicillin-resistant *Staphylococcus aureu*s(MRSA) colonization status among newly admitted newborn in neonatal intensive care unit and risk factors for MRSA colonization.

## Methods

All patients admitted to the neonatal intensive care unit(NICU) at a university hospital in Seoul, Korea from January 1 to December 31, 2008. Data were collected by electronic medical record and retrospectively reviewed. Prevalence was assessed and risk factors for MRSA colonization were compared.

## Results

A total of 599 patients were admitted to the NICU during the study period. Surveillance culture for MRSA was done within 48 hours after admission. MRSA was isolated from 139(23.3%) of the patients. Among the 361 inborns, Fifty-three percents of the patients were admitted via emergency room, and then 36.1% via outpatient department, 19.3% via nursery room, 0.8% via operating room, and 0% via delivery room. Among the 238 outborns, the prevalence of MRSA was vary from 0% to 80% based on the birth place. In multivariate analysis comparing MRSA colonized patients, over 2,500g of admission weight(P= 0.037; odds ratio[OR]=3.2; 95% confidence interval[CI]=1.1-9.6), over 1day of admission age(P=0.014; OR=4.4; 95% CI=1.4-14.5), admission via outpatient department(P=0.040; OR=11.4; 95% CI=1.1-116.9) and admission via other hospital(P=0.002; OR=31.7; 95% CI=3.4-292.7) were independent predictors of MRSA colonization.

## Conclusion

These results showed that MRSA colonization is typically considered to be associated with the neonatal care process than the neonatal status. National support and community participation is necessary to prevent MRSA transmission more effectively.

## Disclosure of interest

None declared.

